# Advances in Growth Factor Delivery for Bone Tissue Engineering

**DOI:** 10.3390/ijms22020903

**Published:** 2021-01-18

**Authors:** Érica Resende Oliveira, Lei Nie, Daria Podstawczyk, Ahmad Allahbakhsh, Jithendra Ratnayake, Dandara Lima Brasil, Amin Shavandi

**Affiliations:** 1 Food Engineering Department, School of Agronomy, Universidade Federal de Goiás, Campus Samambaia, Goiânia CEP 74690-900, Goiás, Brazil; erica_resende_oliveira@ufg.br; 2 College of Life Sciences, Xinyang Normal University, Xinyang 464000, China; 3 Department of Process Engineering and Technology of Polymer and Carbon Materials, Faculty of Chemistry, Wroclaw University of Science and Technology, 4/6 Norwida Street, 50-373 Wroclaw, Poland; daria.podstawczyk@pwr.edu.pl; 4 Department of Materials and Polymer Engineering, Faculty of Engineering, Hakim Sabzevari University, Sabzevar 9617976487, Iran; a.allahbakhsh@hsu.ac.ir; 5 Department of Oral Sciences, Faculty of Dentistry, University of Otago, Dunedin 9016, New Zealand; jithendra.ratnayake@otago.ac.nz; 6 Food Science Department, Universidade Federal de Lavras, Lavras CEP 37200-900, Minas Gerais, Brazil; dandara.brasil@estudante.ufla.br; 7 BioMatter Unit—École Polytechnique de Bruxelles, Université Libre de Bruxelles, Avenue F.D. Roosevelt, 50—CP 165/61, 1050 Brussels, Belgium

**Keywords:** tissue engineering, drug delivery, biomaterials, polymer composites, bone regeneration, growth factor, bone morphogenetic protein, bioscaffold

## Abstract

Shortcomings related to the treatment of bone diseases and consequent tissue regeneration such as transplants have been addressed to some extent by tissue engineering and regenerative medicine. Tissue engineering has promoted structures that can simulate the extracellular matrix and are capable of guiding natural bone repair using signaling molecules to promote osteoinduction and angiogenesis essential in the formation of new bone tissues. Although recent studies on developing novel growth factor delivery systems for bone repair have attracted great attention, taking into account the complexity of the extracellular matrix, scaffolding and growth factors should not be explored independently. Consequently, systems that combine both concepts have great potential to promote the effectiveness of bone regeneration methods. In this review, recent developments in bone regeneration that simultaneously consider scaffolding and growth factors are covered in detail. The main emphasis in this overview is on delivery strategies that employ polymer-based scaffolds for spatiotemporal-controlled delivery of both single and multiple growth factors in bone-regeneration approaches. From clinical applications to creating alternative structural materials, bone tissue engineering has been advancing constantly, and it is relevant to regularly update related topics.

## 1. Introduction

Nonhealing chronic bone tissue defects represent a major problem in healthcare. Despite numerous reports [1,2], there is still a growing need to identify new high-impact compounds for bone tissue regeneration applications. A current approach for bone tissue engineering is based on scaffolds that release growth factors (GFs) required for bone regeneration. A bone scaffold is a 3D matrix that allows for and stimulates the attachment and proliferation of osteoinductive cells on its surface. An ideal scaffold should be biocompatible and should degrade with time to allow new bone deposition; it also should have suitable mechanical properties for load-bearing with proper architecture in terms of porosity and pore sizes for cellular infiltration and angiogenesis, and the ability to control the delivery of bioactive molecules and drugs [3,4,5,6]. Table 1 summarizes recent studies on growth factor-based bone tissue engineering.

Different factors that promote tissue growth have been found at the skeletal damage site and have a physiologic role in healing bone fractures. Osteoinductive GFs such as platelet-derived growth factors (PDGFs), bone morphogenic proteins (BMPs), insulin-like growth factors (IGFs), transforming growth factors (TGFs-ß), and vascular endothelial growth factors (VEGFs) have presented great application potentials in bone healing and osteogenesis for regulating cell behavior, including recruitment, migration, adhesion, proliferation, and differentiation (Table 2) [7,8,9].

Biomechanical stability and biological activity that furnishes an appropriate background for new bone formation are the basis for triumphant GF therapy in bone tissue engineering [9]. Thus, understanding GF biological features, action mechanisms, and delivery strategies are vital for scientists and surgeons.

Several in vivo and clinical studies showed that incorporating GFs into polymer carriers/scaffolds such as gelatin, chitosan, alginate, chitosan, collagen, and hyaluronic acid improved bone healing [2,10,11,12,13]. Among the different carrier materials, absorbable collagen sponges can be used as carriers not only for recombinant human bone morphogenetic protein 2 (rhBMP-2) but also for BMP-9 [14] and BMP-7 [15]. However, this protocol is still limited due to the effective delivery of GFs to tissue, such as release sustainability, stability, inflammation, and ectopic bone formation [16].

A very short duration of action and systemic toxicity by over-release have prevented GFs from being developed into effective regenerative treatments [17]. To circumvent the side effects (i.e., edema), it is foremost important to attain a controllable and sustained release of GFs [18]. Alternatives such as tissue transplantation procedures exist (allograft) but frequently have poor regenerating results, and a better option is needed. Although there is vast applicability for bone bioscaffolds, grafting extracellular matrix (ECM)-derived functional groups to the scaffold is an up-and-coming potential approach for biomaterial design [18]. Successful trials had in common the presence of a control vehicle, which categorically suggests that an effective therapeutic effect is achievable through spatiotemporal management over the targeted area and factor bioactivity [19,20,21].

Emerging and trailblazing materials that modulate the biological presentation of GFs are promising analeptic agents to aid in treating diseases [18,22]. This review considers various biomaterial polymer carriers and GF systemic delivery systems investigated to help the regeneration and repair of bone tissue. In the next sections, general approaches to the strategic use of these factors are discussed in detail and some specific applications for these factors in regenerative medicine are covered. Currently designed approaches and investigated essential topics related to polymer-based carriers for particular technical objectives are also addressed.

### 1.1. Growth Factors Roles in Bone Tissue Engineering

Studies have shown the projected perspectives of tissue engineering. However, triumphant translations into the clinical application are still restricted owing to the shortfall of delivery systems with optimal signaling. Thus, engineers and scientists are promptly developing biomimetic drug delivery systems that can take advantage of reproducing signaling molecules released by the native ECM during healing or regeneration processes. Designed drug delivery systems aim to provide control over the localization, time, and kinetics of the release pattern of signaling molecules such as GFs according to the drug chemical properties and specific biological mechanisms [23].

**Figure 1:**
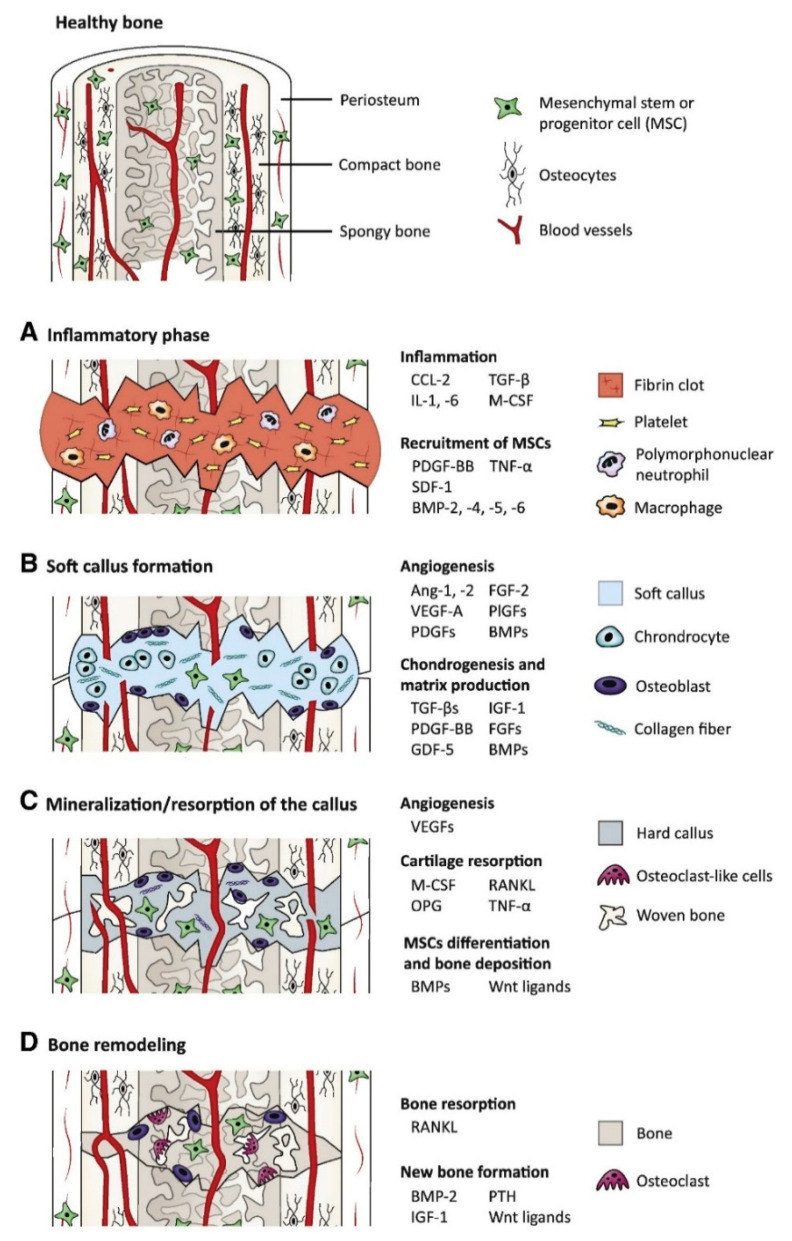
The main growth factors that are relevant to the bone-regeneration process: the bone-regeneration process is addressed in four overlapped, different phases of inflammation (phase **A**), soft callus formation (phase **B**), mineralization and resorption of the soft callus (phase **C**), and bone remodeling (phase **D**) (BMP: bone morphogenetic protein, FGF: fibroblast growth factor, GDF-5: growth/differentiation factor 5, IGF-1: insulin-like growth factor 1, PTH: parathyroid hormone, M-CSF: macrophage colony-stimulating factor, OPG: osteoprotegerin, PDGF: platelet-derived growth factor, PlGF: placental growth factor, RANKL: receptor activator of nuclear factor κB ligand, SDF-1: stromal cell-derived factor 1, TGF-β: transforming growth factor β, TNF-α: tumor necrosis factor α, and VEGF: vascular endothelial growth factor) [18].

Biological signal molecules have a crucial function in modulating cellular activities and tissue regeneration. Bioactive compounds such as GFs are proteins that regulate many aspects of cellular function, including survival, proliferation, migration, and differentiation [24], and have an essential contribution to ECM synthesis [25]. Due to the essential role of GFs in controlling cellular functions and their ability to directly promote and engineer tissue regeneration, a wide range of GFs has been studied and tested for therapeutic applications [26], including bone regeneration [27]. Fibroblast GFs (FGFs), VEGFs, IGFs, TGFs-β, PDGFs, and BMPs are the main groups of GFs associated with bone regeneration [28]. Proteins such as recombinant human BMP-2, BMP-4, BMP-6, BMP-7, and BMP-9 that are currently used in clinical trials are expected to stimulate local bone regeneration by signaling the differentiation of mesenchymal stem cells (MSCs) into osteoblasts [29,30]. Currently, special focus has been given to BMP-2 and 7, as they were approved by the FDA (Food and Drug Administration) for bone-regeneration applications [31]. For instance, BMPs have been shown to elicit new bone formation both at the bone defect site and at heterotopic sites in a large number of species. The process of bone regeneration encompasses the initial inflammatory phase, soft callus formation, mineralization, and bone remodeling [32]. The different phases of bone regeneration engage multiple GFs in specific spatiotemporal patterns (Figure 1).

In the bone-repair process, angiogenesis precedes the onset of osteogenesis. A combination of angiogenic (VEGF), cell recruiting (platelet-derived growth factor (PDGF)), and osteogenic (BMPs) GFs has been designed and demonstrated a synergistic effect that is more beneficial to bone repair than any GF delivered alone [33]. This synergism was also demonstrated through the immobilization of FGF-2 and BMP-2 in administered ratios on the surfaces of gelatin nanofibers to promote bone regeneration [34]. BMPs stimulate the osteogenic and chondrogenic differentiation of mesenchymal cells and play a significant role in structural development throughout the body, having a wide range of functions, including embryogenesis and regulation of cells widely expressed in several tissues [35]. BMPs also display sites for N- and O-glycosylation, allowing for an increase in BMP stability and half-life in the body and determination of the specificity of receptor coupling [36,37]. The integration of stem cells with BMP-2 to promote healthy bone regeneration has demonstrated great new bone formation, fast healing, and callus remodeling [2]. The therapeutic effect of collagen particles combined with BMP-2 with the collagen-binding domain has been shown to reconstruct vertebral laminar defects [38]. That being said, BMP-GFs have an osteoinductive potential for orthopedic clinical practice for the treatment of bone tissue regeneration.

At the surgical site, a specific delivery system should use GFs to exert and maintain biological activity in a controlled fashion and to avoid any systemic diffusion. Therefore, a delivery system is imperative to stabilize GFs and to provide long-term sustained release for in vivo efficacy. Understanding the biomolecular processes during the healing of injured organs is essential for developing GF-based therapeutics for tissue regeneration. An aspect of the natural healing process is the continuous delivery of GFs throughout recovery, avoiding a high variability of GF concentration at the target tissue and rapid clearance [39].

**Figure 2:**
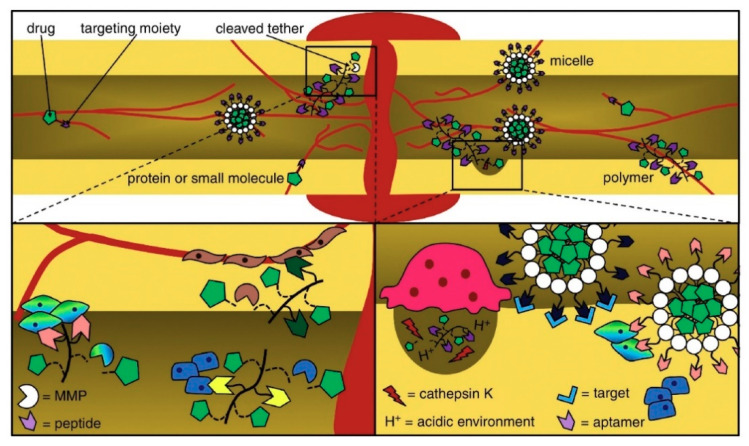
Peptides and aptamers are targeting moieties used to deliver drugs to bones through carriers that transit or infiltrate the blood stream and come out after targeting. The delivered drugs are metabolized owing to a pH media variation or via matrix metalloproteinases (MMP) and enzymes [48].

A successful delivery system can deliver GFs to areas besides the target spot through surgery. This system can maintain enough bioactive factors during the time needed to promote osteogenesis and low fundamental doses to prevent side effects due to supraphysiological GF doses [40]. Delivering osteogenic and angiogenesis-promoting GFs [41,42] together can be a feasible alternative to reestablishing vascularized bone tissue, which is a defying task in bone tissue engineering. Delivering distinct GFs simultaneously, overall, enhances the innate bone-healing process [43]. Local alendronate administration to control β-tricalcium phosphate (β-TCP) resorption and the induction of bone formation by rhBMP-2 were attempted [44]. However, the administration of rhBMP-2 promoted a burst release and reduced osteoclastic resorption of β-TCP induced by rhBMP-2, resulting in decreased bone formation. Supraphysiological delivery of bone tissue GFs resulted in the development of heterotopic bone and other side effects [45]. Octacalcium phosphate/collagen (OCP/Col) can also be used as a carrier system to reduce the rhBMP-2 effective dose. Bien et al. [46] implanted OCP/Col discs impregnated with rhBMP-2 (about 0.25 μg) in mice calvarial bone defects that resulted in no bone formation. Therefore, it is paramount to deliver an effective amount of drug to the defect site. To overcome the mentioned drawbacks, GF carrier systems may play a key role in determining GF bioactivity. Drug injection affecting the whole system or grafting of a polymeric scaffold modified with a bone-targeting moiety delivers a nonintrusive approach for site-specific or targeted therapy [47]. By changing the type of receptor and cell to which the GF binds, the same GF can convey different instructions (Figure 2). Moreover, the same receptor can translate different messages depending on the intracellular transduction pathways, which can differ from one cell type to another.

### 1.2. Scaffold Properties for Bone Tissue Engineering

**Figure 3:**
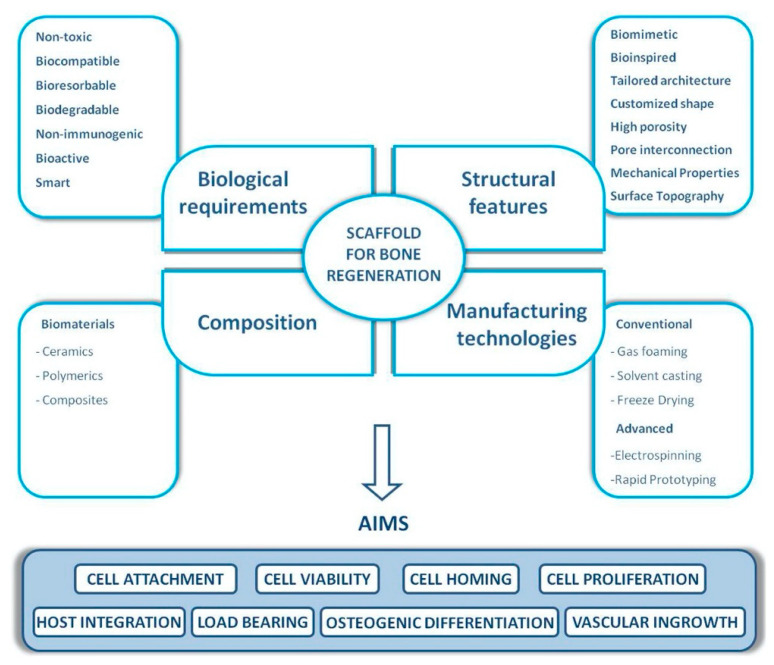
The main biological and structural properties, common compositions, and manufacturing technologies of bone tissue engineering scaffolds [61].

Evidenced by the wide range of inflammatory, osteogenic, and angiogenic factors involved in all bone tissue regeneration processes, these processes can be directly related to biomolecular and cellular processes [47]. GFs’ therapeutic roles can be effectively attained by reaching the damaged tissue site without losing their bioactivity and remaining in the specific site over the healing process [49]. Thus, it is foremost important to develop release technologies to administer the release of signaling molecules in space and time. A proper GF material should be able to manage GF delivery system kinetics to realize tissue formation by efficiently loading the factor and by stimulating protein presentation to the surface of cells (Figure 3). GF release profiles involve prolonged, multifactorial, or sequential releases depending on the type of molecule being delivered and the biological demands [50]. An effective carrier for GFs not only should allow site-specific delivery but also should strengthen the infiltration of cells. Moreover, GFs should accurately load the bioactive factors to allow strong carrier/protein associations [51]. Ultimately, the fabrication process should be straightforward and viable and should maintain the bioactive status of the integrated protein. Overall, scaffold-based GF delivery aims to orchestrate cell response by connecting the transmission of signals from the cells to the kinetics of bone damage healing. Tissue engineering scaffolds not only should prevent ectopic bone formation by facilitating fast infiltration of host cells from margins to the center of the scaffold but also should present low immunogenic and antigenic responses [52]. When GFs are loaded into a scaffold, the incorporation levels and the kinetics that encompass sustained therapeutic doses should be achieved [53,54]. Moreover, the scaffold should degrade into harmless products at a rate that provides the host tissue with a successfully developed mechanical stability [55]. Considering that bones are composed of miscellaneous components such as hydroxyapatite (HA) mineral, organic components (type I collagen, lipids, and non-collagenous proteins), and water [56,57], this combination of materials likely allows the biological activity of scaffolds and their bio-architecture to be accomplished [54]. The bioactivity of tissue engineering scaffolds can also be improved by integrating compounds that correlate organs and cells at the cellular organizational level [58] and, therefore, lead to osteoconduction (bone cell ingrowth), osseointegration (steady attachment to the tissue defect), osteoinduction (stimulation of immature cells into osteogenic ones), and vascularization [59]. Due to the versatile roles of natural bone in the body, bone tissue engineering scaffolds should present several different characteristics to effectively function as a bone scaffold [60]. The main structural characteristics (such as high porosity, high mechanical properties, and tunable architecture), common compositions (polymers, ceramics, and composites), biological requirements (including nontoxicity, biocompatibility, low immunogenic response, and bioactivity), as well as conventional and advanced manufacturing methods (including freeze-drying, electrospinning, and solvent casting) for bone tissue engineering scaffolds are listed in Figure 3.

Such structures provide initial biomechanical support to the implanted tissue until cells can develop a proper ECM to support the regeneration process. It is expected that the scaffold is gradually degraded and metabolized during the formation, deposition, and organization of the ECM, allowing for the tissue to be reestablished with the same or improved function. Thus, such scaffolds are engineered to be biocompatible, biodegradable, and porous to assure vascularization, to show mechanical reinforcement, and to allow functional and bioactive responses [62]. Bone grafts should be biocompatible, bioresorbable, osteoconductive, osteoinductive, structurally similar to bone, easy to use, and cost-effective. The biomaterial properties and features determine the cascade of events that take place at the site of bone healing [63]. The biomaterial should be dissolved or absorbed by the body to be considered bioresorbable. Biomaterials directed for tissue regeneration should degrade continuously in vivo besides filling the defect [64]. As discussed, polymeric, ceramic, and composite scaffolds have been widely considered for bone tissue engineering scaffolds. Although the incorporation of metal nanoparticles in polymeric scaffolds is known to effectively improve scaffold mechanical properties [65,66], the application of metal scaffolds for GF delivery is limited due to the low biodegradability, high rigidity, limited integration to the host tissue, and infection possibility of metal scaffolds [61]. Moreover, compared to polymeric scaffolds, porous metallic scaffolds mostly can only be manufactured through complex procedures, such as electron beam melting [67], layer-by-layer powder fabrication using computer-aided design strategies [68], and extrusion [69], which further limits their architecture design and application in GF delivery [61]. To avoid compromising the function and structure of new bone, the degradation rate of bone biomaterials should match the growth rate of the new structure [70]. Osteoconductive materials allow vascularization of the tissue and further regeneration in addition to building its architecture, chemical structure, and surface charge. Osteoinduction is related to the mobility and propagation of embryonic stem cells as well as cell differentiation [63]. Briefly, scaffolds should present reduced immunogenic and antigenic responses whilst making host cell infiltration easier. Loading efficiency and release kinetics that account for controlled delivery of a therapeutic dosage of GFs are necessary; additionally, scaffolds should degrade into non-harmful substances in a way that the tissue can regenerate its mechanical properties [71,72].

## 2. Polymer Scaffolds for GF Delivery

**Figure 4:**
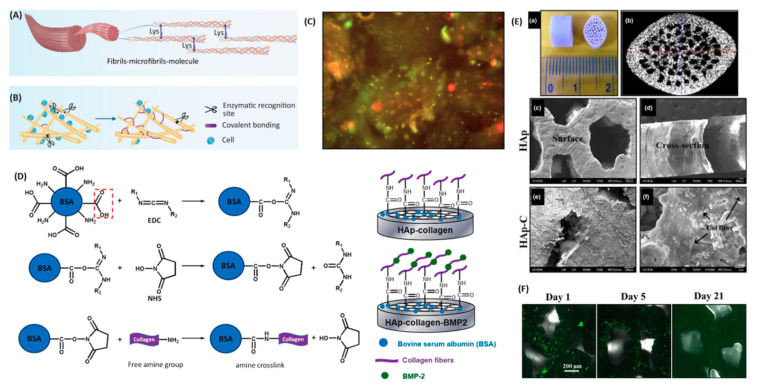
(**A**) Natural crosslinking of collagen (head-to-tail); (**B**) the intermolecular crosslink of collagen allowing for the protection of collagen from enzymatic degradation; (**C**) live/dead cell viability assay of PDLSCs (periodontal ligament stem cells) performed in collagen powder before implantation and 24 h after incubation showing that cells in green are alive; (**D**) mechanism of reaction to modify a collagen scaffold functionalized with hydroxyapatite and BMP-2, and modified scaffolds; (**E**) hydroxyapatite scaffold (**a**) micro-CT pore structure (**b**), surface morphology (SEM) (**c**), cross-sectional morphology (SEM) (**d**), and hydroxyapatite and collagen scaffold (SEM) (**e**,**f**); and (**F**) fluorescent-stained images of a collagen-hydroxyapatite-modified scaffold detecting BMP-2 after 1, 5, and 21 days [75,80,81].

Collagen is the most studied natural polymer for bone tissue engineering scaffolds, as this biopolymer integrates about 90 wt.% of natural bone ECM proteins [73]. Collagen can actively facilitate the osteogenic process of bone progenitor cells through a series of alpha–beta integrin receptor interactions and, as a result, can promote bone mineralization and cell growth [50]. The inter- and intra-chain crosslinks of collagen are key to its mechanical properties which maintain the polypeptide chains in a tightly organized fibril structure. Although collagen has a direct impact on bone strength, this biopolymer has mechanical properties that are insufficient for creating a load-bearing scaffold. Furthermore, the mechanical and degradation properties of collagen can be customized through the process of crosslinking [74] by forming composites [75], as shown in Figure 4. It is, therefore, often combined with more robust materials to create composite scaffolds. As the major inorganic component of bone, HAp has frequently been combined with collagen in composite scaffolds. The mechanism of reaction involved in collagen surface modification and BMP-2 functionalization of 3D hydroxyapatite [76] scaffolds is displayed in Figure 4.

Linh et al. [77] conjugated collagen and BMP-2 to the surface of a porous HAp scaffold. The composite scaffold showed higher compressive strength (50.7 MPa) compared to the HAp scaffold (45.8 MPa). Moreover, the delivery system in this composite scaffold structure more efficiently induced adipose-derived stem cell osteogenic differentiation than in HAp or HAp-collagen (without BMP incorporation) structures. HAp-collagen has been shown to be very effective in healing critical-sized bone damage in a rodent model after HAp shows high affinity to the GFs (BMP-2 and VEGF) used in combination to regenerate vascularized bone tissue [78]. This affinity allows for localized delivery of GFs at the targeted defect site.

In addition to collagen, other natural biopolymers such as silk fibroin can also be effective in bone tissue engineering applications [79,80]. Silk fibroin is a fibrous protein produced by silkworms and spiders with outstanding mechanical characteristics, high biological compatibility, and an adjustable degradation rate that can support cell differentiation [81,82] and, thus, versatility in processing. Composite silk fibroin (Antheraea mylitta) scaffolds were reinforced with functionalized carbon nanofiber to deliver BMP-2 and TGF-β1 [83]. Loaded scaffolds presented a sustained GF release profile; strong adhesion; and the development, propagation, and differentiation of MSCs into osteoblasts. Moreover, composite structures exhibited high compatibility with a targeted immune system, as evidenced by minimal pro-inflammatory cytokines release, both in vitro and in vivo. By depositing HAp on the silk fibroin nanofibrous matrices, enhanced mechanical resistance and a resourceful BMP-2 and TGF-β1 delivery system were observed [84] that induced propagation and differentiation of osteoblasts at the early stages of healing [82].

**Figure 5:**
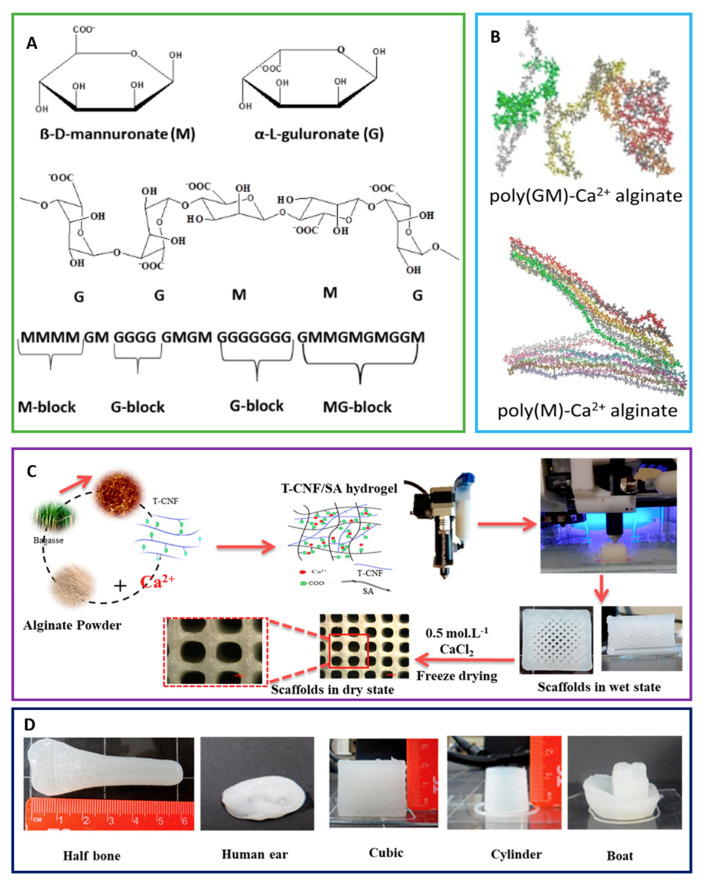
(**A**) Schematic representation of alginate showing the structure of mannuronate (M) and guluronate (G), and the chair conformation and the sequence of M block and G block arrangement in alginate are shown. (**B**) Poly (GM)-Ca^2+^ alginate and poly(M)-Ca^2+^ alginate are displayed. (**C**) The fabrication process for 3D-printed scaffolds from TEMPO-oxidized cellulose nanofibril/sodium alginate hydrogels is shown. (**D**) Scaffolds printed in different forms and designs from optimal TEMPO-oxidized cellulose nanofibril/sodium alginate hydrogel formulation are shown [92,93,95].

Sodium alginate is a linear anionic binary polysaccharide that consists of α-L-guluronic acid (G units) and (1-4)-linked β-D-mannuronic acid (M units) segments. This biopolymer is mostly obtained from widely available seaweeds, which makes it a great candidate for a diverse range of tissue engineering applications (Figure 5). Consecutive G (GGGGGG), M residues (MMMMMM), and alternating M and G residues (GMGMGM) compound the blocks [85]. The composition ratio of these monomers (M/G ratio) and the sequence of monomers in the polymeric backbone determine the final properties of alginate [86]. Alginate is capable of forming stabilized scaffolds through divalent cations crosslinking (i.e., Ca^2+^) due to the anionic nature that allows alginate complexation to these cations [87]. This modification opens avenues for a multitude of medical applications as it overcomes the hurdles faced by using native alginate, such as degradation rate and stability under aqueous conditions [88]. A partially cross-linked TEMPO-oxidized cellulose nanofibril/alginate hydrogel was used to fabricate 3D-printed scaffolds using Ca^2+^ crosslinking [89]. Alginate matrices were conjugated to calcium phosphate scaffolds to achieve a programmed GF delivery [90]. PDGF and BMP-2 were released sequentially with a 3-day PDGF to BMP-2 delivery overlap. It has been suggested that the sequential programming of PDGF to BMP-2 delivery promoted the differentiation of MSCs into osteoblast phenotypes and increased cellular infiltration.

An alternative to overcoming the challenges faced by composite biomaterials is the use of cellulose and other nature-derived polymers once vast manufacturing approaches and sources are available [91]. Cellulose occurs naturally and is an accessible polymer after it is refined from lignocellulose or synthesized from bacteria [92]. Hydrogels with specific structures and diverse functionalities that have biomedical applications can be prepared by manipulating the functional groups in the structure of cellulose and its derivatives (methylcellulose, carboxymethylcellulose, and hydroxypropylmethylcellulose) [93]. Nonetheless, cellulose hydrogels show restricted mechanical attributes that hold back their utilization in hard tissue applications. To surpass this limitation of cellulose-based scaffolds and to build on the functional properties for hard tissue application, mineralization of cellulose hydrogels with HAp and other materials has been actively investigated in recent years [90,91,92,93,94,95,96,97,98,99,100]. Bacterial cellulose was successfully combined with HAp to deliver BMP-2 [94]. The system kinetics was studied in vitro and showed a gradual release of BMP-2 and mineralization spots. Also, BMP-2-loaded aligned electrospun cellulose nanocomposite nanofibers were studied for in vivo bone regeneration in a rabbit model [95]. The results suggest a slight difference between the GF release of aligned and random scaffolds. The aligned scaffold delivered the GFs (0.74 μg/mm^2^) slightly slower than the random scaffold (0.76 μg/mm^2^) after seven days.

Chitin–chitosan is a nitrogen-containing polysaccharide-based biopolymer group derived from diverse natural raw materials such as fungi, crustaceans, and insects [96,97]. Chitin and chitosan are structurally similar to glycosaminoglycans (GAGs, the major component of the bone ECM), which make them suitable biopolymers for tissue engineering scaffolds [96,97,98]. Chitin used in combination with chitosan/poly(vinyl alcohol) to fabricate nanofibers showed enhanced mechanical properties and offered osteoblast cell growth with HAp biomineralization [99]. Chitosan nanoparticles loaded with BMP-2 were dispersed into collagen hydrogel and added to the scaffolds. The system showed active osteoinduction through the controlled delivery of GFs [99]. Drug delivery systems using β-tricalcium-phosphate/gelatin containing chitosan-based nanoparticles [100] and dextran sulfate-chitosan microspheres [101,102] were designed to promote the sustained delivery of BMP-2 for bone tissue regeneration. Both systems showed that alginate composite scaffolds were able to attain the controlled release profile of GFs and to act as a mechanically and biologically compatible framework with prominent osteoinductive activity.

Recent studies have suggested GAGs as potential biomaterials for tissue engineering application, as this biopolymer predominantly exists in the ECM, has low immunogenicity, and can perform strong interactions with GFs [103]. The structural composition (degree of sulfation and polymer length) of GAGs are varied and determine the precise performance of GAGs. Cell-binding motifs, native-like mechanical properties, bone mineralization-specific sites, and robust GF binding and signaling capacity are among the GAG properties [104,105]. Notwithstanding, investigations on GAGs as molecules for engineering tissue scaffolds have been conducted as of late. GAGs isolated from mammalian sources such as heparin [47,106], heparan sulfate [76,107], chondroitin sulfate [108,109], keratan sulfate [110], and hyaluronic acid [111,112] (non-sulfated) are the most widely explored in regeneration medicine. Strong ionic interactions are expected between GAGs and proteins. Among the GAGs, hyaluronic acid is the predominant GAG in the skin whereas chondroitin sulfate is the major GAG found in bone. GAGs interact with residues that are prominently exposed on the surface of proteins. Clusters of positively charged basic amino acids on proteins form ion pairs with spatially defined negatively charged sulphate or carboxylate groups on GAG chains. The main contribution to binding affinity comes from ionic interactions between the highly acidic sulphate groups and the basic side chains of the protein. Despite incomplete understanding of the interactions between cells and ECM, namely, at the molecular level, it is known that GAGs modulate the adhesion of progenitor cells and their subsequent differentiation and gene expression. These regulatory roles are related to the GAG ability to interact with GFs and to protect GFs from proteolytic degradation, increasing the half-life of GFs. For instance, during osteogenesis, heparan sulfate provides matrix-bound or cell surface-bound reservoirs for specific binding proteins, including GFs such as BMPs [47]. In vivo BMP-2 retention can be improved via heparin microparticles (HMPs). HMPs can improve the safety profile of scaffold-based BMP-2 delivery systems and, consequently, can reduce the heterotopic ossification. Moreover, these microparticles can improve the spatial localization of bone formation in large bone defects. Overall, GAGs play an important regulatory role in the development and regeneration of skin and bone tissue by performing complex effects on skin and bone cells at all stages of their differentiation, including the attraction and adhesion of precursor cells, their subsequent differentiation, their activity and immune responses, and their interactions with other proteins. Thus, GAGs are part of a new genesis of biomimetic biomaterials.

## 3. Encapsulation, Incorporation, and Related Delivery Strategies

**Figure 6:**
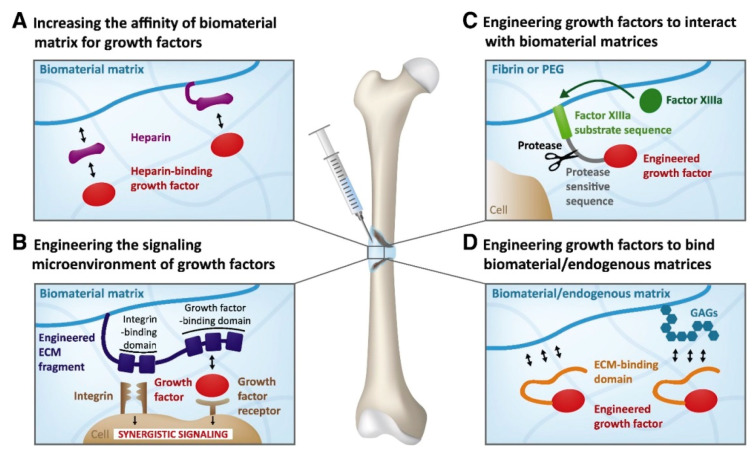
Schematics of delivering systems of growth factors based on the extracellular matrix (ECM) ability to protect growth factors from degradation and to avoid the formation of concentration gradients (a regulatory mechanism): (**A**) a biomaterial matrix covalently incorporates or co-receives a heparin/heparin-mimetic modified matrix, which binds the growth factors. (**B**) Receptor (i.e., integrin and growth factor) synergistic signaling through the addition of a fibronectin fragment that has both receptor domains is shown. (**C**) A growth factor is recombinantly introduced for the factor XIIIa substrate sequence. (**D**) A growth factor is recombinantly produced for incorporation into the ECM-binding domain that interacts with ECM proteins and/or glycosaminoglycans (GAGs). As a result, the growth factor can bind endogenous ECM or biomaterial matrices constituted of natural ECM proteins such as fibrin and collagen [18].

A large number of techniques have been presented and employed to manage the release kinetics of GFs entrapped in scaffolds. A majority of successful methods is based on encapsulating GFs in a degradable polymeric network [23], which can gradually release the GF from the scaffold into the defect site (Figure 6). Using this process, the therapeutic dosage release can be extended much longer than currently available rapidly releasing scaffolds [28,113]. In this section, recently developed strategies and techniques for the fabrication of GF-incorporated scaffolds with a sustained release rate of GFs are covered. Such a sustained release of these biomolecules can provide a more physiologically relevant environment for the promotion of bone regeneration. Direct injection and systematic local supplementation of the scaffold/GF system can lead to rapid in vivo degradation, deactivation by enzymes, and a short half-life in the physiological environment [114]. The lack of dynamic and targeted kinetics of GF molecules has shown burst releases and supraphysiological dosages [115] leading to the likelihood of untimely and unwanted effects and has instigated the need to address such limitations. Nano-delivery systems providing an artificial ECM for cell attachment and penetration while keeping a 3D network to allow facilitated and guided tissue regeneration have been explored [116].

**Figure 7:**
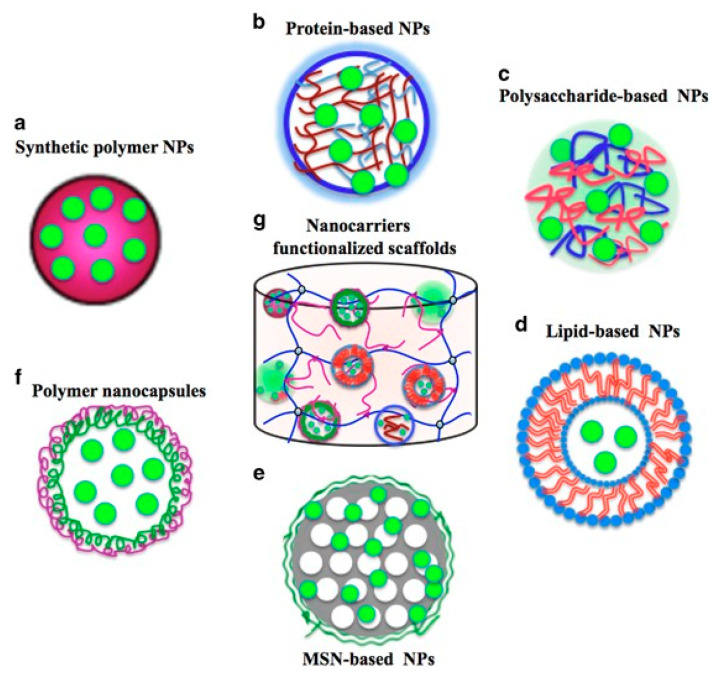
Different nanocarrier types applicable for the encapsulation and release of growth factors (GFs) (**a**–**f**) and a modified scaffold functionalized with nanocarriers for encapsulating GFs (**g**) [121].

Physical entrapping processes for the incorporation of bioactive molecules in polymer networks can also strongly affect the performance of these systems. Different techniques are available to entrap drug molecules in the structure of scaffolds, which facilitate their contact with migrating cells and regulate cell behavior (Figure 7). Surface presentation entitles site-specific drug delivery and could narrow their potential off-target side effects [117]. The two key methods for introducing biomolecules to the scaffold surface are physical adsorption and chemical conjugation. The first approach allows for diffusion-based release by adsorbing GFs into a substrate. The latter involves covalent/noncovalent bonding of GFs straight to the surface of the substrate. Furthermore, it is possible to attach GFs to linkers, which are molecules that connect the GFs and the immobilizing surfaces [47,106,118,119,120].

### 3.1. Physical Adsorption

From a technical point of view, physical adsorption can be considered the most straightforward method for embedding biomolecules into polymer scaffolds [117]. Physical adsorption can be obtained by integrating biomolecules into a polymer matrix before its gelatinization [122] or by immersing the preformed scaffold in a protein solution. It usually depends on the interactivity amongst the biomolecules and scaffold surface, such as electrostatic interactions, hydrogen bonding, or hydrophobic interactions [123], and on the biomolecule structure [40]. Delivery of GFs to the defect site depends on scaffold porosity, temperature, pH media, the salt concentration of the solute, and the relationship between the protein and substrate. Thus, GF retention relies on its appropriate immobilization on or absorption into the substrate [124]. Surface characteristics such as wettability, roughness, surface functionalities, charge density, and surface charge are some material properties that can affect the physical adsorption of biomolecules on the surface of polymer scaffolds [117]. Physical immobilization of GFs is an easy to accomplish technique in mild conditions and, thus, has raised much interest. Besides, technological readiness, reasonably priced reagents, and maintenance of bioactivity are some of the advantages of GF physical immobilization. On the other hand, inefficient retention of stable soluble protein, a lack of spatial distribution, and release administration can be observed [75]. Notwithstanding the disadvantages, physical immobilization stands as the most common method for attaining GF immobilization [123].

GF adsorption on the defect site has to be steady and localized, and a GF–receptor interaction must occur to activate signaling cascades, inducing osteoblast proliferation, to effectively allow tissue regeneration [125]. Accordingly, an equilibrium between anchored adsorption on the substrate and protein activity protection must be attained [126]. The properties of the scaffold can be preserved using this method, and it does not shatter the bioactivity of GFs. Nevertheless, matrix–factor interaction mechanisms including electrostatic interactions, ECM affinity, or hydrophobic interactions can affect the release profile of GFs [127]. Physical adsorption can be achieved through surface adsorption, encapsulation, and layer-by-layer techniques. BMP-2 was adsorbed on a series of nano-textured HAp surfaces which were substantially important in the liaison of BMP-2 dynamic behavior [127]. Compared to the HAp-flat model, the HAp-1:1 group (ridge vs. groove = 1:1) was able to incorporate BMP-2, which showed fewer changes in its conformation. Moreover, the HAp-1:1 group showed high cysteine-knot stability through adsorption/desorption processes, indicating that nano-textured HAp surfaces can better incorporate BMP-2 molecules through adsorption and can aid in BMP-2 biological activity. Alginate microbeads were surface condensed with heparin through polyelectrolyte complexes (diethylaminoethyl-dextran (DEAE-D), poly-l-ornithine, and poly-l-arginine) to provide a delivery system for BMP-2 [128]. The authors observed distinct release profiles for each of the systems designed. Although most microbeads can release about 60% of the adsorbed BMP-2 throughout three weeks, the DEAE-D-based microbeads can present a fast GF release of 2 days, showing structured posterolateral spinal bone formation in a rat model. The pattern of GF release from noncovalent systems at the diffusion- and degradation-dependent levels, including biomolecule desorption, scaffold degradation, and protein–scaffold interaction failure mechanisms [48]. The diffusion-dependent release follows first-order kinetics and is conditioned to the GF size and related to the scaffold pore size. Diffusion-dependent release is restricted when the scaffold pores are smaller than the hydrodynamic radius of the incorporated protein [129]. Control over the release rate can be possible by modifying the material degradation rate and mechanism [130,131,132]. Increasing the electrostatic attraction between GFs, such as BMP-2 and TGF-β, and the scaffold matrix can also improve the loading efficiency [122].

Surface functionalization via physical adsorption has the advantage of being a simple and gentle procedure accompanied by limited damage to fragile structures and biomolecules. However, biomolecule binding to scaffold surfaces can be relatively weak [133]. The scaffold surface can be further modified to improve its affinity for drug molecules. Heparin has been used to modify the scaffold surface to improve GF binding to the scaffold, allowing for the controlled release of BMPs [134], PDGF [135], and VEGF [136] in tissue regeneration-related studies. The surface coating is known widely to improve the GF scaffold affinity. The scaffold surface can be physically and chemically coated via proteins such as gelatin, heparin, and fibronectin to modify the scaffold surface with specific biological sites to immobilize GFs [137]. Different superficial immobilizing models including physical adsorption, covalent grafting, and heparin-binding (self-assembled monolayer) to fabricate BMP-2-immobilized surfaces distinctly influenced the loading capacity and osteoinduction in vivo and in vitro [138]. In the in vitro studies, osteoinduction was noted in the covalently grafted model, followed by the physically adsorbed model when the saturated dosage of BMP-2 was applied. In contrast, the physical adsorption model was more efficient when inducing osteogenesis when a similar amount of BMP-2 was used (120 ng) for each model. Heparin scaffold strengthened BMP-2 and BMP-2 receptor recognition and weakened BMP-2 attachment to its competitor, demonstrating heparin’s selectivity in inducing in vivo bone tissue differentiation. Specifically, BMP-2 cell recognition efficiency can be handled via an orientation that can be a potential design target to achieve BMP-2 delivery vehicles with improved therapeutic efficiencies. One of the first techniques used to build a delivery system to release multiple GFs is direct adsorption; nonetheless, the release kinetics in a controlled or programmable manner has been proven to be challenging in addition to having a loss of bioactivity [139]. Thus, alternative maneuvers have been used to address these bottlenecks. Electrostatic interactivity between polyelectrolytes with opposite charges and GFs are used to deliver functionalized polymer overlays on a myriad of surfaces [121]. This approach is called layer-by-layer. Notably important to protein delivery, the layer-by-layer method requires facile aqueous baths which potentially preserve soluble protein activity, as the method does not need to use harsh organic solvents [140]. During tissue regeneration, different GF profiles are present, and the multilayer biotechnology is an open venue that allows for building GF carriers with appropriate delivery kinetics that are able to simulate those GF profiles. For instance, a polydopamine multilayered coating was used to associate BMP-2 and VEGF, where BMP-2 was bound onto the inner layer and VEGF was bound onto the outer layer [141]. The authors reported a more rapid VEGF delivery succeeded by a gentle and more continuous release of BMP-2. Additionally, angiogenic and osteogenic gene expression assessment indicated a collaborating effect between the GF-loaded scaffolds and the co-culture (human bone marrow-derived mesenchymal stem cells (hMSCs) and hEPC) conditions.

A brushite/PLGA composite system to control the release of PDGF, TGF-β1, and VEGF was designed to promote bone remodeling [142]. PDGF and TGF-β1 were delivered more rapidly from brushite cement compared to VEGF in a rabbit model where approximately 40% PDGF and TGF-β1 were delivered on the first day. In the next six following days, the release rates were reduced by approximately 5.5% per day, and a total release of 90% was observed after three weeks. In contrast, scaffolds incorporated with VEGF were more efficient in tailoring the release profile by controlling it (7%/day in the first week; 1.2%/day for three weeks), with a total release of approximately 80% within two months. Therefore, GF-loaded microspheres built into scaffolds allow for an uninterrupted and long-lasting release of GFs from scaffolds.

### 3.2. Chemical Conjugation

**Figure 8:**
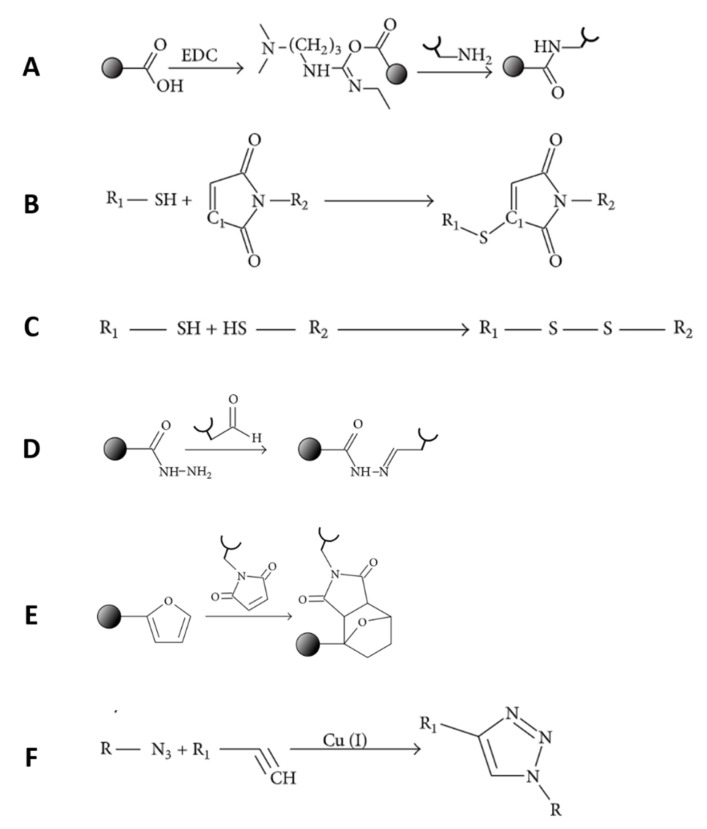
Covalent bond formation between growth factor and carrier: (**A**) amide group, (**B**) thioether group, (**C**) disulfide group, (**D**) acetyl-hydrazone group, (**E**) polycyclic group, and (**F**) click chemistry [155].

Chemical conjugation, or covalent bonding, offers prolonged and more stable drug molecule presentation than the physical adsorption method [23,143]. For this process, the scaffold surface needs to be activated with functional groups that can then conjugate with drug molecules through proper chemical reactions [122] (Figure 8). Nonetheless, most of the scaffolds applicable in bone tissue engineering are degradable and deficient in reactive groups [144]. The primary approaches for functionalization of scaffolds are modification after fabrication and incorporation of GFs before fabrication. However, the fact that the conjugation reaction may modify the biomolecule conformation and result in the loss of bioactivity is an important issue [145]. For instance, covalently grafted (chemical coupling process) BMP-2 may affect ectopic bone formation due to unwanted self-crosslinking of BMP-2 during the reaction [146]. Therefore, many drugs are pre-modified (e.g., conjugation to a PEG spacer) [147] and drug mimics (GF peptide mimics) [148] are utilized. Various bioconjugation reactions have been investigated, with reactions conducted in aqueous solution or under mild reaction conditions being particularly favorable. Copolymerization and chemical/physical reactions between active groups of scaffolds and GFs are widely used to incorporate biomaterials and cargos [149]. Amidation, esterification, and click reactions are some of the commonly used reactions for this purpose [150]. Suboptimal doses of BMP-2 (2.5 μg) can be chemically conjugated on a collagen scaffold via a crosslinker, Traut’s reagent, and a cross-linker (4-(N-maleimi-domethyl) cyclohexane-1-carboxylic acid 3-sulfo-N-hydroxysuccinimide ester sodium salt) to obtain a controlled GF delivery system for bone tissue regeneration with no ectopic formation [151]. Moreover, in rat models, co-treatment with stromal cell-derived factor-1α (SDF-1α) and the suboptimal dose of BMP-2 chemically interacted on the surface of collagen scaffolds can induce higher levels of ectopic bone formation compared to physically interacted systems. Moreover, Zhang et al. [144] reported that a collagen membrane chemically conjugated with SDF-1α can promote new bone and microvessel formation significantly compared to a system with SDF-1α physical adsorption. Thiol-ene click reaction was used to conjugate a BMP-2 mimicking peptide (P24) onto a nanofibrous scaffold [152] to guide tissue formation. As a chemical reaction may modify the GF molecular structure and create a loss in bioactivity [153], mimicking biomolecules are encouraging strategies in GF release from scaffolds and unveil their functionality [154] within tissue regeneration. The scaffold showed the bioactivity and osteoinduction of rabbit bone marrow-derived MSCs. Udomluck [34] developed a GF delivery system based on heparin chemically conjugated to decellularized bone particles to allow for electrostatic tethering of PDGF. Bone particles with tethered GF promoted bone mineral deposition by adipose-derived stem cells in vitro and, hence, bone formation mediated by stem cells in vivo within murine critical-sized calvarial defects. Wang et al. electrospun a scaffold of porous gelatin nanofibers to improve the bone growth and to imitate the function of natural ECM for sustained release of multiple GFs. The scaffold system was coated with HAp in a simulated body fluid solution and surface-functionalized with avidin to facilitate binding with biotinylated GFs such as BMP-2 and FGF-2 at different ratios [75]. Multiple GFs were successfully conjugated onto the functionalized surface of the scaffold by controlling the FGF-2/BMP-2 ratio. The release profiles were compared with those of physical adsorption, and a more continued and controlled release for avidin-biotin pairing was observed. The delivery of various GFs and the overlayer out of HA-nanofiber synergistically optimized bone healing, which was substantiated by the incremented osteogenic gene marker expression. Therefore, the nanofiber scaffold is an up-and-coming osteoconductive vehicle to deliver multiple GFs in a sustained manner.

Controlled and sustained release of BMP-2 and VEGF built-in silk fibroin/nanoHA scaffolds via chemical and physical covalent bonding, respectively, was observed [75]. VEGF promoted the formation of new blood vessels at the beginning stages of bone healing, while the spatiotemporal release of BMP-2 led to in vitro and in vivo osteogenic differentiation. The in vivo trial in a rat model resulted in complete bone formation in calvaria defects after 12 weeks. These results suggested that the combination of appropriate doses (BMP-2: 300 ng per scaffold and VEGF: 20 ng per scaffold) of multiple GFs incorporated into an ideal scaffold have a synergistic effect on vascularized bone regeneration. Thus, GF covalent bonding to scaffolds has advantages in the management of long-term release systems compared to the physical adsorption method.

### 3.3. Spatiotemporally Controlled Delivery of GFs

**Figure 9:**
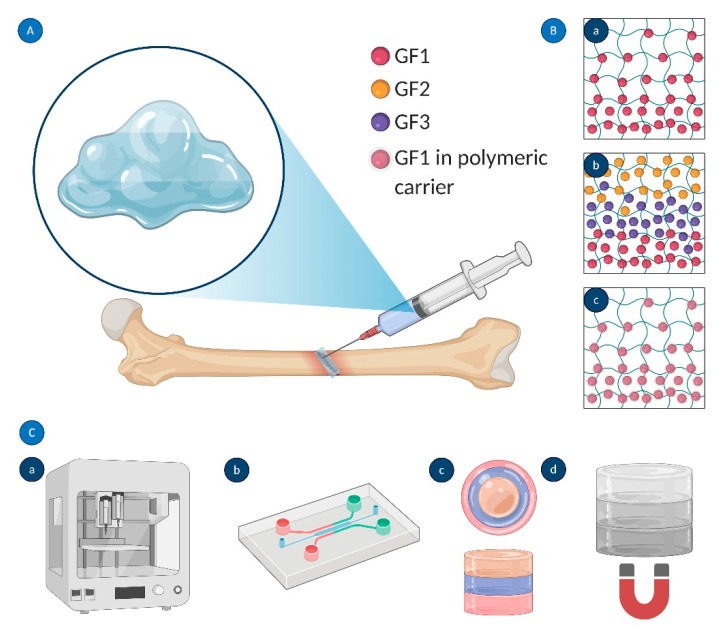
Engineered GF gradients: (**A**) injection of graded biomaterials for bone regeneration; (**B**) strategies used to create GF gradients within hydrogels: (**a**) concentration gradient of a single biomolecule (GF1), (**b**) sequential delivery of three different biomolecules (GF1, GF2, and GF3), and (**c**) encapsulation of biomolecule(s) in polymeric micro- and nanocarriers; and (**C**) methods for graded biomaterial fabrication: (**a**) 3D bioprinting, (**b**) microfluidics, (**c**) layer-by-layer scaffolding, and (**d**) magnetically (electrically) driven distribution of GFs. Created using Biorender.com.

Biochemical gradients in the cellular microenvironment are known to drive a variety of physiological processes including bone repair [156]. The major role of growth factor gradients in bone formation is to stimulate cells to migrate in the direction of gradually increasing concentrations of signaling biomolecules (chemotaxis) [157,158]. The neighboring cells sense the changes in signal concentrations and respond accordingly. The cellular response and subsequent bone formation depend on bone morphogenic protein concentration and occur only if the BMP threshold dose is achieved [23]. To address those challenges, implantable polymeric, the biomolecule-delivering systems, and carriers are engineered to balance between growth factor release and retention to reach the optimal dose of cues for stimulation of bone regeneration. By releasing BMPs, the delivery device induces cells to migrate towards the injury while the retained factors promote bone formation within the defect [105]. Bone tissue itself is a functionally and structurally graded system [159]. Bone remodeling, on the other hand, involves seven sequential phases (quiescence, activation, resorption, reversal, formation, mineralization, and termination), each regulated locally by the expression and release of growth factors in a sequential manner [39,160]. The highest effectiveness of bone formation in vitro is expected to be achieved in bone tissue-mimicking systems. So far, many biomaterials have been designed to provide spatiotemporal control over growth factor delivery to enhance osteogenesis. A proper design of delivery systems with an ability to locally control over spatial distribution and sustained release of the biological agents may prevent the side effects and toxicity to the surrounding healthy tissues [161]. For example, James et al. recognized major side effects associated with the clinical use of BMP-2, which includes inflammatory and wound complications, ectopic bone formation in the surrounding soft tissues, and bone resorption due to osteolysis [162]. Most drug-releasing systems use natural polymers (e.g., collagen and alginate) as matrices for immobilization of GFs and other biologically active molecules. However, those polymer-only scaffolds may suffer from rapid and uncontrolled GF sequestration; thus, more advanced strategies are now being developed. These include novel materials and devices that allow for the sequential release of multiple growth factors and other chemical cues. Figure 9 demonstrates the current approaches for the generation of chemical gradients within hydrogels. Graded materials can be designed to have either single (Figure 9(Ba)) or multiple (Figure 9(Bb)) gradients of biologically active molecules.

One of the strategies for sequential GF delivery assumes the incorporation of various nanoparticles with encapsulated growth factors into polymeric scaffolds [49] (Figure 9(Bc)). Several studies have reported the fabrication of PLGA (poly(lactic acid-co-glycolic acid)) capsules loaded with different growth factors and then immobilized in hydrogel matrices. Sequential VEGF delivery and BMP-2 were achieved by the inclusion of alginate microcapsules embedded with GF-containing PLGA NPs into the collagen matrix [163]. Despite its complexity, this system allowed for the effective transport of biomolecules and their functional synergism in bone regeneration. Wang et al. [164] utilized microencapsulation in a hydrogel matrix for the generation of a single concentration gradient and a dual reverse gradient of bone morphogenetic protein 2 (rhBMP-2) and insulin-like growth factor I (rhIGF-I) to induce osteochondral differentiation of hMSCs. Microsphere GF carriers fabricated from silk and PLGA were further incorporated in silk fibroin or alginate scaffolds. The hMSCs were differentiated into osteoblast-like (cuboidal) and chondrocyte-like (spherical) cells along the concentration gradients. Because silk microspheres turned out to be more efficient GF vehicles than PLGA microcapsules, the authors proposed a silk-based platform for delivery of multiple biomolecules that allows for regulation of the spatial control over distribution and temporal control over sequestration of GFs. In a study by Yilgor et al., wet-spun chitosan and chitosan-PEO scaffolds were embedded with PLGA and poly(3-hydroxybutyrate-co-3-hydroxyvalerate) (PHBV) nanocapsules containing BMP-2 and BMP-7, respectively [165]. The sequential delivery of the growth factors enhanced alkaline phosphatase activity, which was an early indicator of MSC differentiation into chondroblasts and osteoblasts.

Hettiaratchi et al. developed a BMP-2-delivering system based on the strong affinity interactions between heparin microparticles (HMPs) and bone morphogenic proteins embedded within an alginate/polycaprolactone scaffold. By binding BMP-2 to HMPs, the authors reduced the rate of biomolecule diffusion of BMP-2 by generating its long-term gradient and by controlling spatial localization [105]. In another study, heparin-conjugated superparamagnetic iron oxide nanoparticles (heparin-SPIONs) were used to generate a magnetically driven biochemical gradient of BMP-2 within a cell-laden agarose hydrogel. The BMP-2 concentration gradient governed the spatial osteogenic gene expression to form robust osteochondral constructs with hierarchical microstructure from low-stiffness cartilage to high-stiffness mineralized bone [166].

Recent technological advances in biomanufacturing have enabled the biofabrication of biomaterials with differentially arranged growth factor gradients. These advanced techniques include 3D bioprinting, microfluidics, layer-by-layer scaffolding, and techniques that utilize magnetic or electrical fields to distribute biomolecules within scaffolds (Figure 9C) [166,167]. Layer-by-layer (LbL) scaffolding has been utilized to create multilayered scaffolds embedded with several growth factors. In such systems, each layer is cured individually and contains a different biomolecule or concentration. The separation of biologically active agents into different shells is based on the interactions between scaffolding material and a cue. The LbL technique allows sequential delivery of various bioagents and creates a spatial gradient of growth factors release. Shah et al. designed a polyelectrolyte multilayer system formed by a layer-by-layer (LbL) method to deliver multiple biologic cues in a controlled, preprogrammed manner. The gradient concentration of growth factors was created by sequential depositing polymeric layers laden with BMP-2 directly onto the PLGA supporting membrane, followed by coating with mitogenic platelet-derived growth factor-BB-containing layers. The released GFs induced bone repair in a critical-size rat calvaria model and promoted local bone formation by bridging a critical-size defect [33]. Freeman et al. [168] utilized a 3D bioprinting technique to print alginate-based hydrogels containing a spatial gradient of bioactive molecules directly within polycaprolactone scaffolds. They created two distinct growth factor patterns: peripheral and central localizations. To enhance the bone repairing process of large defects, the authors combined VEGF with BMP-2 in a properly designed implant. The structure contained vascularized bioink (VEGF) in the core and osteoinductive material at the periphery of the PCL scaffold. Proper control over the release of the signaling biomolecule was achieved by combining alginate with laponite, the presence of which slowed down the release rate in comparison to the alginate-only biomaterial. This approach was found to enhance angiogenesis and bone regeneration without abnormal growth of bone (heterotopic ossification). In Kang et al., FGF-2 and FGF-18 were successively released from mesoporous bioactive glass nanospheres embedded in electrospun PCL scaffolds. The nanocomposite bioactive platform stimulated cell proliferation and induced alkaline phosphate activity and cellular mineralization leading to bone formation [169].

All currently used strategies for engineering and fabrication of graded tissue scaffolds for bone regeneration are guided by the same principles: (1) to mimic native bone tissues and to follow the ordered sequence of bone remodeling, (2) to generate complex multifunctional gradients, (3) to control the spatiotemporal distribution and kinetics of biological cues, and (4) to be easily generated by accessible and reproducible techniques.

## 4. Considerations for using GFs in Bone Tissue Engineering

### 4.1. Toxicity

Growth factors have shown great potential in bone regeneration. However, their clinical applications are limited due to the following reasons: short biological life in physiological conditions due to rapid degradation and deactivation, high cost, and side effects [170]. There are other safety issues around the use of GFs in bone regeneration, including bony overgrowth, immune responses, inflammatory reaction, nerve damage, breathing problems, cancer, and osteoclastic activation [171,172,173,174]. BMPs were adopted by many surgeons as a replacement for autologous bone grafts following FDA approval in 2002. However, clinical safety issues were brought to light with several serious complications reported regarding the use of BMPs postoperatively, which included oedema leading to dysphagia and dyspnea, bone graft resorption, and osteolysis [18,175,176]. Growth factor effects are dose-dependent. Several studies have shown that minimally effective doses are needed to be determined above a certain threshold for bone formation as bone formation cannot be further enhanced. Dose-dependent bone healing was observed when IGF-1 was loaded into a sheep femoral defect. New bone formation was observed for 30 and 80 μg but not for 100 μg IGF-I, which resulted in roughly the same effect as that for 80 μg [177,178]. Aspenberg et al. [179] reported that the application of excessive doses could provoke or inhibit bone formation. Therefore, it is important to customize the dosage for each factor and delivery system for successful GF delivery [180].

The use of appropriate delivery systems can considerably enhance the safety and efficacy of GF therapies. When GFs are used for bone repair, the materials which are prepared for the delivery system must be nontoxic and biodegradable [181]. The main role of a delivery system for bone repair is to retain the GF at the defect site for bone regeneration and to restrain the drug from excessive initial dose release [174]. Hollinger et al. showed that, for BMPs, if delivered in a buffer solution, clearance is rapid and less than 5% of the BMP dose remains at the defect site. However, when BMPs were delivered with either gelatin foam or collagen, an increase in retention ranging from 15% to 55% was observed [182]. Adverse effects have been mainly associated with systematic GF release, whereas localized delivery is significantly safer. Nevertheless, when high doses of rhBMP-2 were administered locally, heterotopic bone and bone-cyst formation was reported during defect healing in dogs [183]. Furthermore, osteoclastic resorption was also reported, and in some cases when large doses were applied, bone resorption occurred [184]. However, human studies using rhBMP-2 have not demonstrated systemic toxicity.

### 4.2. Cost

Besides the side effects, the cost-effectiveness of GFs for bone regeneration applications is also under debate. The translation of GFs is narrowed by their delivery issues, side effects [185], and low cost-effectiveness [186]. A study conducted by Dahabreh et al. showed that the average cost of treatment with BMP-7 was 6.78% higher than that with autologous-iliac-crest-bone grafts. Furthermore, 41.1% was related to the actual price of BMP-7 [187]. Another study showed that the use of rhBMP for spinal fusion surgery would increase the cost to the UK NHS by approximately £1.3 million per year and that the total estimated cost of using BMP for spinal fusion is about £4.2 million per year in the UK [188].

## 5. Current Strategies and Future Trends

The bioactivity of GFs plays a vital role in bone regeneration. Even after several in vivo and in vitro studies, the ideal dosage of GFs applied for bone regeneration remains uncertain [189]. When administered without optimal delivery systems, burst release kinetics and rapid clearance of GFs from the injury site are major challenges in terms of safety and cost-effectiveness. In recent years, using a combination of scaffolds and GFs has become an increasing trend in bone regeneration. To be effective, GFs should reach the injury site without losing any bioactivity and must remain at the target site over the therapeutic time frame. Therefore, designing biomaterials as various delivery systems or carriers allowing dose reduction, controlled release kinetics, and precise localization in situ and promoting enhanced cell infiltration is an effective strategy in improving bone tissue engineering [50,190]. Furthermore, the carrier biomaterial must load each GF efficiently, must encourage the presentation of proteins to cell surface receptors, and must promote robust carrier–protein assembly [191,192]. Finally, fabricating the carrier should be simple and feasible and should be able to preserve the bioactivity of the GF for prolonged periods.

To meet the requirements of GF delivery, several scaffold-based approaches such as physical entrapment of GFs within the scaffold, covalent or noncovalent binding of the GFs to the scaffold, and the use of micro or nanoparticles as GF reservoirs have been developed [49]. Covalent binding reduces the burst release of GFs, allows GFs to have the prolonged release, and improves the protein-loading efficiency [49]. However, the limitations of covalent binding include high cost and difficulty in controlling the modification site, blocking of the active sites on the GF, and thus interference with GF bioactivity [193]. Noncovalent binding of GFs to scaffold surfaces involves the physical entrapment or bulk incorporation of GFs into a 3D matrix [49]. The simplest method of GF delivery is often considered to be protein absorption, and it is the method used by current commercially available GF delivery systems [194]. Varying certain material properties such as surface wettability, roughness, surface charge, charge density, and the presence of functional groups are used to control the protein absorption to scaffolds. Unlike, covalent binding and noncovalent binding systems are characterized by an initial burst release of the incorporated GFs, followed by a degradation-mediated release which depends on the scaffold degradation mechanism. The release mechanism includes degradation of the scaffold, protein desorption, and failure of the GF to interact with the scaffold [138]. Therefore, the delivery of GFs from noncovalent bound systems are both diffusion- and degradation-dependent processes. The major drawbacks of noncovalent protein absorption in scaffolds are poor control of release kinetics and loading efficiency [194]. Therefore, new strategies focusing on altering the material’s degradation and improving the loading efficiency have been investigated. One such example is increasing the electrostatic attraction between GFs such as BMP-2 and the scaffold matrix [138,193]. Moreover, different fabrication methods such as hydrogel incorporation, electrospinning, and multilayer film coating have been employed to fabricate scaffolds with noncovalently incorporated GFs. A study conducted by Sahoo et al. showed that electrospinning could be used to prolong GF release from scaffolds and sustained GF release, which positively influences stem cells [195].

Hydrogels are a common GF delivery strategy as they can act as a scaffold or as protein releasing matrices [196]. Studies have found that hydrogels can demonstrate a preliminary burst release followed by sustained GF release over 28 days in systems with high GF-loading concentrations [197]. Moreover, GFs can be encapsulated in nanoparticles and then incorporated into scaffolds to reach more precise control over GF release and can achieve a long-term sustained GF release profile [75]. There are several advantages in encapsulating GFs within nanoparticles. The advantages include ensuring protection from enzymes in vivo, allowing for prolonged protein retention, and obtaining a certain degree of control over the protein release profiles [190,198]. Other advantages include improving osteointegration, osteoconduction, and osteoinduction by mimicking the complex hierarchical structures of the natural bone and environment, high drug loading capacity, large surface, and small size [114].

## 6. Conclusions

In this review paper, recent developments in fabricating scaffolds for GF delivery in bone tissue regeneration were discussed. Despite progress covered in this paper, more work is required to develop biomaterials that are porous and mechanically strong, that can present controlled degradation, and that match the rate of new bone formation. Well-known side effects of direct GF injection lead to the clinical need for developing delivery systems with controlled GF delivery. Among the different available strategies, GF encapsulation in the structure of scaffolds can be considered a promising method to control the release kinetics of GFs and to fabricate scaffolds with improved characteristics. The GF/scaffold release system should mimic the coordinated fracture repair pathway in practical applications. Moreover, delivery systems with the capability of delivering multiple GFs in a targeted manner could promote the inflammation, angiogenesis, and osteogenesis phases of bone formation.

## Figures and Tables

**Table 1:** Studies on growth factor-based bone tissue engineering.

Growth Factor	Material Carrier	Fabrication Method	Delivery Remarks or Mechanism of Action	Application	In Vivo or In Vitro Tests	References
PDGF-BB	β-tricalcium phosphate (TCP) particles	-	Interaction with PDGF receptors stimulates recruitment and proliferation of cells and promotes revascularization.	Distal radius fractures, hindfoot/ankle fusion; healing in hindfoot and ankle arthrodesis	In phase III randomized, controlled trial, 66.5% of PDGF-treated joints and 62.6% of autograft-treated joints showed fusion on computed tomography scanning at 24 weeks postoperatively.	[199,200]
FGF2 + BMP2 VEGF + BMP2	Silica-coated nanohydroxyapatite-gelatin reinforced with poly (L-lactic acid) (PLLA) yarns	GFs dissolved in PBS and loaded onto the scaffolds	FGF2 mainly promoted cell migration, whereas VEGF augmented new blood vessel formation at the defect site.	Promotes vascularisation and bone regeneration in a critical-sized calvarial defect	In in vivo and in vitro tests, VEGF was released for 1 week whereas BMP2 and FGF2 were released for 3 weeks. In vitro studies have shown that the composite matrix degraded partially within 2–3 weeks in the presence of a collagenase enzyme. Release of growth factors was faster in vivo than in vitro. This disparity may be due to a complex in vivo environment containing multiple matrix-degrading enzymes (MMP2 and MMP9), cell types, etc. that are involved in the healing process.	[201]
BMP-2	Polyelectrolyte (PEM) film coating	Polyelectrolyte film loaded with tunable doses of BMP-2 as the osteoinductive surface coating of a hollow PLGA tube	(a) Release owing to the swelling of the film (b) Release due to biodegradability of the film	Triggers fast volumetric bone regeneration via the surface of an implant	(a) Microcomputed tomography and quantitative analysis, and C2C12 cell culture and in vitro BMP-2 bioactivity assay (b) In vivo critical-size femoral defect in the rat: formation of vascularized cortical and cancellous bone (c) The formation of new bone dependent on the dose of BMP-2: higher doses lead to hematoma	[202]
BMP-2 and TGF-β1	Silk protein fibroin reinforced with functionalized carbon nanofiber (CNF)	Facile green aqueous-based	Prolonged-release kinetics; timely growth, attachment, multiplication, and differentiation of mesenchymal and osteoblasts cells	Extracellular matrix for osseointegration	(a) Cytocompatibility of growth factor loaded matrices showed immunocompatibility due to low release of pro-inflammatory cytokines (TNF-α and IL-1β). (b) In vivo analysis of new bone formation within the implants (radiological, μ-CT, fluorochrome labeling, and histological analysis) demonstrated more efficient bone regeneration on loaded scaffolds.	[83]
bFGF (basic fibroblast growth factor)	Porous α-TCP particles	Immobilization on heparin-modified α-TCP by immersion	Stimulation of osteoblast proliferation and differentiation	Mandible cortical bone regeneration	In an in vivo test on a canine model, higher bone mineral content and formation of homogenous cortical bone with Haversian structure dependent on bFGF dosage (optimal dose of 4.2 μg) was seen.	[34,203]
rhBMP-2	Absorbable collagen sponge (ACS) and β-TCP/hydroxyapatite particle (TCP/HAp)	Immobilization on the carrier by immersion	Stimulation of osteoblast proliferation and differentiation	Tooth alveolar ridge preservation	In an in vivo test on a human model, similar bone height and width with no associated deleterious effects were seen.	[204,205]
BMP-2	Alginate and Collagen	Scaffold loading by droplet	(a) Collagen sponges showed initial burst release within a day. (b) Alginate showed a more controlled release.	Regeneration of femoral segmental defects	(a) BMP-2 release in vitro was accelerated from collagen sponge, and loaded alginate induced higher bioactivity. (b) In an in vivo test on a rat model, an alginate scaffold showed higher total bone volume at 12 weeks; heterotopic bone volume was similar for alginate and collagen.	[2,11]
hBMP-2 and hGDF5 (human growth and differentiation factor)	Titanium (Ti)	Coated onto Ti with a smooth surface using heparin-binding interaction	Initial burst release at day 1 followed by controlled release for 30 days	Orthopedic and dental bone formation and osseointegration	(a) An in vitro test showed a high proliferation rate and alkaline phosphatase activity resulting in calcium deposition and gene expression. (b) An in vivo test on a rabbit model showed bone regeneration and osseointegration between the implants and host bone. Bone formation by osteoblasts and bone resorption by osteoclasts was observed through histological analysis	[205,206]
BMP-2 and FGF-2	Gelatin nanofibers	Immobilization on nanofibers through avidin-biotin binding after HAp deposition	A synergism between multiple growth factor delivery and the HAp nanofiber coating stimulated the expression of osteogenic gene markers.	Promotes bone growth and mimics the natural extracellular matrix	Immobilization of FGF-2 and BMP-2 in administered ratios on the surfaces of gelatin fibers resulted in cell proliferation.	[2,34]
PDGF	Poly(ι-lactic acid) (PLLA) nanofibers	Immobilization on PLLA nanofibers coated with biominerals	Osteogenic and endothelial differentiation with gene expression	Vascularized bone regeneration	(a) In vitro. PDGF increased the proliferation of hADSCs (human adipose-derived stem cells). (b) In an in vivo mouse calvarial defect, bone regenerated 42.48% of an area and formed capillaries and arterioles.	[205]
VEGF and BMP-2	nHAp/poly lactic-*co*-glycolic acid microspheres (PLGAs)/chitosan [207] hydrogel	Water-oil-water double emulsion solvent evaporation method (PLGA-loaded microspheres) and immersion (HAp and CS)	Sustained release with early burst release in the first 10 days followed by a steady release of BMP-2 (days 11 and 21) and VEFG (day 11 and 19), and bioactivity preservation	Ossification and vascularization in critical-sized mandibular bone defects	In an in vivo rabbit model, bone defect cavities gradually reduced with time and healed after 12 weeks with callus remodeling.	[2]

**Table 2:** Growth factors used for bone tissue engineering either directly or delivered via a scaffold [119,208,209,210,211].

Name of Growth Factor	Abbreviation	Source	Biological Response	Mechanism of Action	Functions
Bone Morphognetic Proteins	BMP	Mesenchymal Osteoblast Endothelial Chondrocyte	Chondrogenic, osteogenic, and osteoinductive	Bone induction	BMPs are osteoinductive and induce bone formation by causing the migration of MSCs and their differentiation into osteoblast. BMPs do not initiate osteoclast activity.
Fibroblast Growth Factors	FGF	Mesenchymal Osteoblast Chondrocyte Inflammatory Cell Endothelia	Angiogenesis and connective tissue cell proliferation	Angiogenesis, proliferation, and osteogenic differentiation	FGFs induce angiogenesis by increasing osteoblast proliferation and a potent stimulant for wound healing.
Insulin-Like Growth Factors	IGF	Osteoblast Chondrocyte Hepatocyte Endothelial	Anabolic and catabolic effect on osteogenesis	Osteogenic differentiation	IGFs stimulate osteoblast proliferation and bone matrix synthesis. IGFs also stimulate osteoclasts.
Platelet-Derived Growth Factor	PDGF	Platelet Osteoblast Inflammatory Cells Endothelial	Osteoinductive, angiogenesis, and connective tissue cell proliferation	Cell proliferation and vascularization	PDGFs are a key regulator of wound healing/tissue repair and stimulate bone cell proliferation and angiogenesis
Transforming Growth Factor-Beta	TGF-β	Platelet Osteoblast Chondrocyte Endothelial Inflammatory Cells Fibroblast	Osteoinductive, immunosuppression, angiogenesis, andcell growth and differentiation	Osteogenic and chondrogenic differentiation	TGF-β induces proliferation and differentiation of bone by stimulating migration of osteoprogenitor cells and by regulating cell proliferation, cell differentiation, and extracellular matrix (ECM) synthesis and inhibits proliferation and differentiation of osteoclast progenitor cells.
Vascular Endothelial Growth Factor	VEGF	Platelet Osteoblast Chondrocyte	Osteoinductive, chemotactic, and angiogenesis	Angiogenesis	VEGF regulates migration, proliferation, and survival of endothelial cells through nutrient supply from newly formed blood vessels.
